# The Golden Ratio of Gait Harmony: Repetitive Proportions of Repetitive Gait Phases

**DOI:** 10.1155/2013/918642

**Published:** 2013-06-04

**Authors:** Marco Iosa, Augusto Fusco, Fabio Marchetti, Giovanni Morone, Carlo Caltagirone, Stefano Paolucci, Antonella Peppe

**Affiliations:** ^1^Santa Lucia Foundation, IRCCS, Via Ardeatina 306, 00179 Rome, Italy; ^2^Dipartimento di Medicina dei Sistemi, Università di Roma “Tor Vergata,” Via Montpellier 1, 00133 Rome, Italy

## Abstract

In nature, many physical and biological systems have structures showing harmonic properties. Some of them were found related to the irrational number *ϕ* known as the golden ratio that has important symmetric and harmonic properties. In this study, the spatiotemporal gait parameters of 25 healthy subjects were analyzed using a stereophotogrammetric system with 25 retroreflective markers located on their skin. The proportions of gait phases were compared with *ϕ*, the value of which is about 1.6180. The ratio between the entire gait cycle and stance phase resulted in 1.620 ± 0.058, that between stance and the swing phase was 1.629 ± 0.173, and that between swing and the double support phase was 1.684 ± 0.357. All these ratios did not differ significantly from each other (*F* = 0.870, *P* = 0.422, repeated measure analysis of variance) or from *ϕ* (*P* = 0.670, 0.820, 0.422, resp., *t*-tests). The repetitive gait phases of physiological walking were found in turn in repetitive proportions with each other, revealing an intrinsic harmonic structure. Harmony could be the key for facilitating the control of repetitive walking. Harmony is a powerful unifying factor between seemingly disparate fields of nature, including human gait.

## 1. Introduction

Harmony is an essential feature of human gait warranting for efficient and smoothed movements during walking [1–3]. Gait harmony has been defined as the capacity to transfer the symmetry of human body into alternated, synchronized, symmetric, and rhythmic movements [3] by means of intralimb [4], interlimb [5], and lower-upper body [6, 7] coordination. In previous biomechanical studies, harmony was estimated computing the proportion between even and odd harmonics of the body kinematics and/or the proportion between two lower limb kinematics recorded during two consecutive steps, that is, within the same gait cycle [1, 3, 8, 9]. 

**Figure 1:**
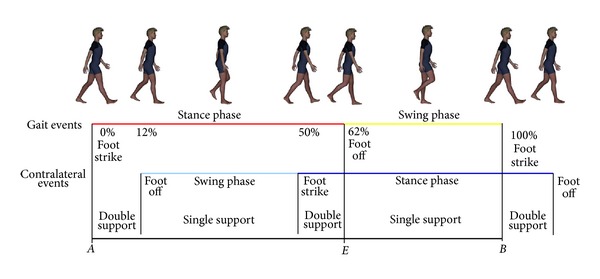
The gait cycle. A schematic representation of gait cycle with stance (red) and swing (yellow) phases showed in the above line, whereas the line drawn in middle reported the gait cycle of the contralateral limb with swing (light blue) and stance (blue) phases. The black line drawn below showed the link with the Euclid's problem to cut a straight line (*AB*) so that the proportion between the shorter part (*EB*) to the longer one (*AE*) is the same as the longer part (*AE*) to the whole (*AB*).

Each gait cycle has been defined as the interval between two repetitions of the same gait event. These events are conventionally identified in two consecutive foot strikes of the same limb [10, 11]. The gait cycle hence comprises a stance phase, in which the foot makes contact with the ground, and a swing phase, in which the foot advances in the air (Figure 1). The stance phase begins with a foot strike and ends with the foot off. Much of the literature agrees that foot off reliably occurs at 60% to 62% of a physiological gait cycle [7, 10–16]. During typical development, stance phase was found directly correlated with walking speed but not significantly dependent on age of children [17]. Also during walking on a steeply inclined surface, the values recorded for stance phase remained in a narrow range (for an inclination of 42° it was found about 60% for descending and about 64% for ascending gait) [14]. This low variability of foot off timing during development and/or among different walking conditions suggests that the proportion between stance and swing (60–62% versus 40–38%) is an invariant of physiological comfortable human gait. The alteration of this proportion is usually identified as a sign of pathological gait [10–16]. Despite it, the reasons for which this proportion is a so reliable parameter of human gait have been poorly investigated.

We have noted that the proportion between stance and swing phase is close to *ϕ*, an irrational number (about 1.618034) already known in ancient Greece as “golden ratio” [18]. This number has been related to the problem reported by Euclid in III century BC to cut a given straight line so that the proportion between the shorter part to the longer one is the same as the longer part to the whole (see Section 2 for details) [18]. The Greek letter *ϕ* was chosen to indicate this number in honor of the sculptor Phidia, who supervised the construction of the Parthenon, the façade of which is a golden rectangle, that is, a rectangle having lengths in the proportion of *ϕ* [18, 19]. Henceforth, mathematicians, physicists, biologists, architects, and artists have been interested in the intrinsic symmetric properties of this number. This number was also called “divine proportion” during the Renaissance, from the title of a book from 1509 by Luca Pacioli and illustrated by Leonardo da Vinci “De divina proportione” [20]. 

From a mathematical point of view, the peculiarity of *ϕ* is related to some harmonic properties of this number. The most important harmonic property of *ϕ* is that the inverse of *ϕ* (i.e., 1/*ϕ*) is equal to *ϕ* − 1. Furthermore, *ϕ* is the asymptotic value towards which the ratio of consecutive terms of Fibonacci sequence converges [18].

A wide variety of seemingly disparate physical and biological systems, such as leaf disposition on plant stems and seed arrangement on flower heads [18, 21], spiral structures of galaxies and mollusks [18, 22], quantum phase transitions [23], nucleotide frequencies [24], and cell [25, 26] and shell [22] growth, show similar harmonic characteristics related to *ϕ*. It was due to the particular geometrical and mathematical properties of this irrational number. In human sciences, *ϕ* has been observed with regard to body proportions [27] and aesthetic preferences [28, 29]. But it has never before been reported as a feature of human motor behavior. 

The aim of this study was to verify the hypothesis, derived from the above observation, that in healthy subjects, stance and swing are in the proportion equal to *ϕ*, implying some important harmonic properties for human gait. 

## 2. Material and Methods

### 2.1. Participants

Twenty-five healthy adults (with no neurological or orthopedic conditions affecting their gait) were enrolled in this study (16 men and 9 women, mean age: 49 ± 19 years old). This study was designed in accordance with the Declaration of Helsinki on experiments involving human beings.

### 2.2. Protocol of Gait Analysis

We analyzed the spatiotemporal gait parameters of all the enrolled subjects. They were asked to walk at their comfortable speed in our laboratory along a linear pathway. Gait analysis [10] was performed using a stereophotogrammetric system (SMART system, BTS Padua, Italy). Six video cameras with a sampling rate of 50 Hz recorded the 3D coordinates of 23 retroreflective markers placed bilaterally on the subjects' skin according to the Davis' protocol [30]. Fifteen of these markers were placed on the pelvis and lower body segments. After calibration, the system showed an error of less than 0.5 mm with regard to spatial accuracy. Temporal accuracy was set by sampling rate and was hence of 0.02 s. Spatiotemporal parameters were averaged between at least 5 trials for each limb of each subject, forming a dataset of 50 mean strides (25 healthy subjects, 2 limbs each one).

### 2.3. The Golden Ratio *ϕ*

As introduced above, in the III Century BC, Euclid described the problem to cut a given straight line so that the proportion between the shorter part to the longer one is the same as the longer part to the whole [18]. If the longer part is 1, the length of the entire segment is an irrational number *ϕ*. It corresponds to the problem to find the point *E* on the segment *AB* such that $\bar{AE}:\bar{AB}=\bar{EB}:\bar{AE}$ (Figure 1). Defining $\bar{AE}=1$; $\bar{AB}=x$; $\bar{EB}=x-1$ the equation becomes 1 : *x* = *x* − 1 : 1 and hence

(1) $$\begin{array}{c} x^{2}-x-1=0, \end{array}$$

the solution of which is as follows:

(2) $$\begin{array}{c} x=\frac{1+\sqrt{5}}{2}≅1.618034\ldots =\phi . \end{array}$$

As stated above, one of the most important harmonic properties of *ϕ* is the following one:

(3) $$\begin{array}{c} \frac{1}{\phi }=\phi -1≅0.618034\ldots \end{array}$$

that is easy to demonstrate the following:

(4) 1ϕ=2(1+5)=2×(1−5)(1+5)×(1−5)=2×(1−5)−4=−1+52=1+52−22=ϕ−1≅0.618034…

### 2.4. Biomechanical Analyses

The hypothesis of this study was that

(5) $$\begin{array}{c} \frac{\text{stance}}{\text{swing}}=\phi . \end{array}$$

Despite the value of 1/*ϕ* falls in the range defined for stance phase in the literature (60–62%, i.e., 0.60–0.62), the fact that stance/swing is not different from *ϕ* needed to be statistically tested on data specifically collected in a sample of healthy subjects of both genders and with a wide age range for taking into account of biological variability.

In the light of geometrical definition of *ϕ*, (5) implies the following:

(6) $$\begin{array}{c} \frac{\text{stance}}{\text{swing}}=\frac{\text{gait}\;\;\mathrm{cycle}}{\text{stance}}=\phi . \end{array}$$

The above equation together with the property reported in (3) implies the following:

(7) $$\begin{array}{c} \frac{\text{swing}}{\text{stance}}=\frac{1}{\phi }=\phi -1=\frac{\text{stance}}{\text{swing}}-1=\frac{\text{stance}-\text{swing}}{\text{swing}}. \end{array}$$

Under the hypothesis of symmetric gait, in which gait phases do not differ significantly between limbs [10, 11], it is possible to consider that stance − swing = stance − contralateral  swing = double  support, corresponding to the sum of the 2 double phases reported in Figure 1. In conclusion, (5), (6), and (7) can be summarized as follows:

(8) $$\begin{array}{c} \frac{\text{gait}\;\;\mathrm{cycle}}{\text{stance}}=\frac{\text{stance}}{\text{swing}}=\frac{\text{swing}}{\text{double}\;\;\text{support}}=\phi . \end{array}$$

Equation (8) can also be elicited in the following system of equations that this study tested:

(9) $$\begin{array}{c} \text{stance}=\frac{100}{\phi },\\ \text{swing}=\frac{100}{\phi^{2}},\\ \text{double}\;\;\text{support}=\frac{100}{\phi^{3}}. \end{array}$$

From a mathematical point of view, all the above equations are strictly dependent on each other. However, from a statistical point of view, all these equations are hypotheses that needed to be tested on collected gait data for verifying the main hypothesis of this study because transitive property of equality cannot be applied to statistics.

### 2.5. Statistical Analysis

A database formed by 50 samples (25 healthy subjects, 2 lower limbs each) was analyzed. Mean, standard deviations, and coefficient of variation (CV = standard deviation/mean∗100) for the entire group were computed. 

So, one-sample two-tailed *t*-test was used to compare values with a single number (such as the three comparisons with *ϕ* reported in (8) or (9) or to test the hypothesis of symmetric gait). In fact, the observation that the value of 1/*ϕ* falls within the narrow range in which foot off occurred during gait cycle did not directly implied that the proportion between stance and entire cycle was not statistically different from *ϕ*. Comparisons between 2 samples (6) were performed by two-tailed paired *t*-test. Comparisons between 3 samples (8) were made by repeated measure analysis of variance. The threshold of significance was set to 0.05. Despite the fact that the high number of tested hypotheses might need a Bonferroni correction on this threshold, being the hypothesis of this study verified if *P* value was higher than the threshold of significance, we adopted a conservative approach without applying any adjustment for multiple comparisons.

## 3. Results

Gait analysis performed on 25 healthy subjects replicated results consistent with the literature [10–14]. The subjects involved in this study walked with a mean speed of 1.14 ± 0.16 m/s.  Table 1 summarizes the average values of their spatiotemporal gait parameters. The lowest coefficients of variation was found for opposite foot strike, occurring at about 50% of gait cycle (CV = 1.55%) and for the foot off (CV = 3.75%) occurred at 61.81 ± 2.32% of gait cycle. In accordance with the hypothesis of symmetric gait, values of opposite foot strike (not expressed in percentage of gait cycle, i.e., 0.501 ± 0.008) were not found significantly different from 0.5 (*P* = 0.476). Foot off value (0.618 ± 0.023) was compared to 1/*ϕ* = *ϕ* − 1≅0.618034…, without finding any significant difference (*P* = 0.974).

**Figure 2:**
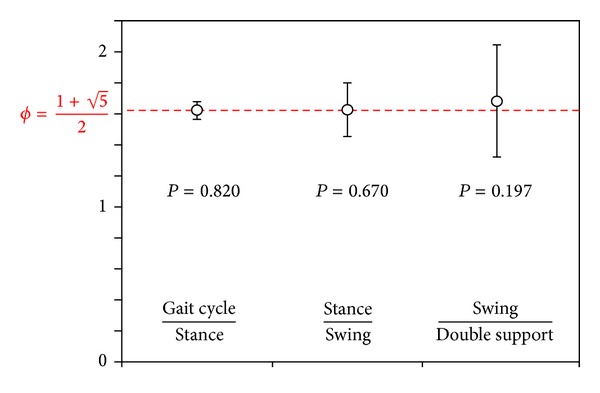
Proportions among gait phases. Mean ± standard deviation of the three ratios computed between phases of the gait cycle and *P* value of comparisons with *ϕ* (the value of which is represented by the red dot line).

To test the main hypothesis of this study (5), the proportion of stance to swing phase (1.629 ± 0.173) was compared to *ϕ*, finding no statistically significant difference (*P* = 0.670). Similarly also the proportion between the entire cycle to stance did not differ significantly from *ϕ* (*P* = 0.820, Figure 2). These results were confirmed by testing (6): no significant differences were found between stance/swing and entire cycle/stance (*P* = 0.793).

The sum of the two double support phases resulted in encompassing the 23.62 ± 13.62% of gait cycle. The ratio between swing and double support resulted in 1.684 ± 0.357 (*P* = 0.197 versus *ϕ*). 

By repeated measure analysis of variance performed to test the hypothesis underlying (8), there were no significant differences between the three ratios (*F* = 0.870, *P* = 0.422).

**Figure 3:**
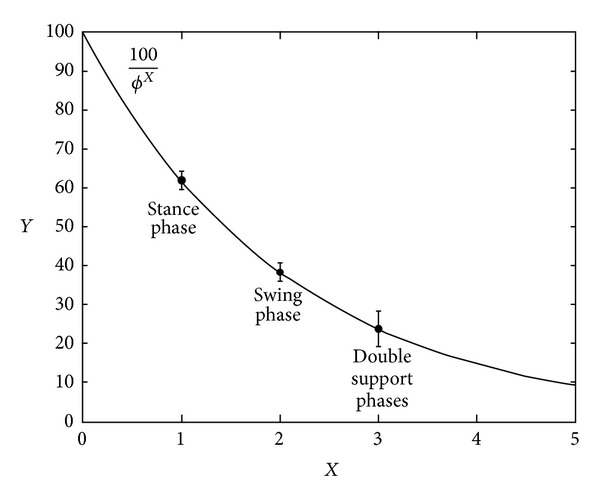
Relationship between gait phases and *ϕ*. Mean ± standard deviation of stance, swing, and double support phases reported as circles on the line derived from the equation *Y* = 100/*ϕ* ^*x*^ in which *X* is the number of progressive cutting of straight line representing the gait cycle (this equation has been obtained by the system of equations reported in Section 2 as (9)).

The differences with theoretical values reported in (9) are graphically shown in Figure 3, and they did not result in statistical significance (*P* = 0.974 for stance versus 100/*ϕ*, *P* = 0.998 for swing versus 100/*ϕ* ^2^, *P* = 0.982 for double support versus 100/*ϕ* ^3^).

## 4. Discussion

The results of this study confirmed the hypothesis that stance and swing phases of physiological human gait are in the proportion of *ϕ*. The values of spatiotemporal gait parameters found in this study are in accordance with those of the literature, but a new insight about their reciprocal proportions has been provided. In fact, this study is the first one putting in relationship harmonic properties of human locomotion with those of *ϕ*. According to the harmonic properties of this irrational number, the repetitive gait phases resulted in turn in repetitive proportions with each other. Even the proportion between swing and the difference between stance and swing (i.e., the sum of the two double support phases) approximated *ϕ*. Double support warrants the upright stability during walking [10, 11]. At the same time, the presence of double support implies the impossibility of a 50 : 50 symmetry between stance and swing. The 50 : 50 symmetry was found for the contralateral foot strike, verifying the symmetric bilateral movements during walking. Previous studies only showed that stance/swing proportion is reduced in fast walking and increased under pathological condition, but the reason for which stance and swing proportion is about 60 : 40 for all healthy people walking at comfortable speed along their lifespan remained unclear, before our results.

Stance to swing ratio can decrease with increasing walking speed, with a theoretical limit of 50 : 50. Stance is <50% and swing >50% in running for the presence of a flight phase that substitutes the double support phase [31]. However, it has been shown that even a high increment in walking speed (90%) implied just a little change in stance phase duration (percentage of stance was found about 63% at 1 m/s and 59% at 1.9 m/s) [13]. The narrow range of foot off timing among different walking conditions, such as the low coefficient of variation found among different subjects in our study (3.75%), suggested a low variability of the stance to swing proportion. 

On the contrary, this proportion was found altered under pathological conditions, such as in patients affected by Parkinson's disease [32], Huntington's disease [33], or muscular dystrophy [7, 15]. A stance to swing proportion of 70% to 30% was found for the affected side of patients with hemiplegia due to stroke [34] or spastic cerebral palsy [35]. A longer stance phase allows for increasing gait stability by means of the extension of double support phases. Hence, a stance between 59% and 70% is a good compromise between functional (fast) and stable walking. The exact proportion of *ϕ* for stance to swing ratio probably entails the harmonic properties found in this study. 

It is generally accepted that the nervous system needs to reduce the complexity of controlling redundant degrees of freedom of bilateral multijoint limbs during locomotion [36]. Harmonic properties of locomotor patterns may facilitate the control of the gait rhythm. In fact, in human mature gait, the temporal activation and deactivation of the involved cortical and subcortical (such as the central pattern generators) structures, as well as the chronological sequence of the movement-related potential, were mapped to specific phases of the gait leg movements [37]. It has been hypothesized that also in humans central pattern generators (CPGs), localized in spinal cord, can sustain the basic locomotor rhythm generating alternating activity of flexor and extensor motoneurons even in the absence of input from higher centers and afferent feedback [38, 39]. The first model depicted CPGs as half-center oscillators (one half for flexor activation and another for extensors) [39]. More recent studies hypothesized that CPGs probably contain these oscillators, but their intrinsic at rhythmogenic properties also depend on other fundamental units [40], on interconnections with afferent feedbacks deriving from muscle spindles and tactile sensors [41], and with the widespread projections of serotonergic neurons of the brain stem [42]. In particular, it has been suggested that simple alternation of flexor and extensor activity can be converted into more complex and adaptable locomotor patterns by hippocampal neurons receiving serotonergic projections from the median raphe nuclei [42]. 

Despite this complexity, oscillators are still at the basis of most of CPG models for producing alternation of flexor and extensor activations [40]. Flexor patterns are mainly involved during swing phase of gait cycle, whereas extensor ones during stance, so it is conceivable that there is a strict link between CPG rhythm and gait one. The differences in sensory and muscle spindles feedbacks between these two gait phases may act reinforcing this link between CPG rhythm and repetitive proportions of stance and swing phases that we found related to *ϕ*. 

Locomotor rhythm can be activated and influenced by the projections of serotonergic neurons of the brain stem innervating spinal cord stepping generator [42]. In patients with Parkinson's disease, a decrease of levels of serotonin in cerebrospinal fluid associated with severe gait and posture disorders has been observed [43]. Stance and swing phases of patients with Parkinson's disease had been reported to be in proportion of 68% and 32% of gait cycle [32], that is, with a ratio of about 2.12, far from the value of *ϕ*. Stance/swing ratio in pathological conditions such as in Parkinson's disease and the possible effects of pharmacological and rehabilitation intervention on this ratio should be specifically investigated in further researches.

Differently from subjects affected by Parkinson's disease, the locomotion of healthy subjects is highly adaptable and context dependent. These adaptations seemed to be mainly related to spatial output and muscle activations, whereas the basic temporal architecture of locomotor primitives seems to be relatively conserved across healthy subjects in many different conditions [38, 44]. Recently, four locomotor primitives, derived from lower limb electromyography and related to spinal cord neural networks, were found in humans similarly as in other animals [45]. Despite the authors did not link these primitives directly to the phases of gait cycle, it is possible to observe that each one of these four primitives had a peak in a specific phase of gait cycle. In fact, first harmonic has a peak during first double support phase, second harmonic during stance, third one during the second double support phase, and the fourth harmonic during swing. This idea can also be supported by the studies reporting that the gait harmony can be estimated by the ratio between even and odd harmonics of body kinematics [1, 3]. Furthermore, four basic muscular activation components remain invariantly timed in walking such as in running with respect to the beginning of the stance phase, supporting the idea of a fundamental role played by gait phase proportion in muscular activation and vice versa [44]. In human newborns, two instead of four locomotor primitives have been identified [45]. They are probably related to extensor and flexor patterns typical of the involuntary stepping. The absence of any real gait phase related to support during stepping reflex hence implies specific changes in locomotor patterns, confirming a strict relationship between muscular patterns and (simulated) gait phases, even in newborns. On the other hand, similar results have already been found in rats: olivary neurons discharge rhythmically at frequencies closely matching step cycle [46]. Analogously, in cats, the locomotion activity of more than 90% of neurons of motor cortex are modulated in the rhythm of strides [47]. 

In general, therefore, it seems that CPGs produce basic alternated activation of extensor and flexor muscles related to stance and swing phases, respectively. Hence, it is likely that harmonic properties of stance and swing phases are an expression of harmonic rhythm generated by central patterns, and probably supported by a reciprocal inhibition and by afferent sensory signals. But also the control of upper limb movements can benefit from harmonic properties of temporal structure of gait. Arm swinging is helpful for reducing angular momentum and vertical displacement of body center of mass during gait [48]. Cervical segments of spinal cord contain generators of oscillatory patterns for coordinating upper limb movements during walking, according to the hypothesis of distributed CPGs for controlling human locomotion [38].

The involuntary control of locomotion can be favored by this temporal harmony. The repetitive proportions of gait phases enlightened in this study may act as an attractor for an efficient motor control in which four different limbs, more than ten joints, and many different muscles should be controlled at the same time. On the other hand, it was even found that the spontaneous walking rhythm could prevail over voluntary reproduction of an external rhythm [49]. This spontaneous and intrinsic rhythm of walking could favor the locomotor control such as metrics favors memory, making poems or songs easier to remember and repeat.

## 5. Conclusions

The importance of this study is not related to providing new spatiotemporal gait data but to providing a new insight of well-known walking data. Our results are strengthened by reliable (low coefficient of variation recorded between subjects) data consistent with those of the literature. In the light of our results, further studies should investigate the development of a harmonic mature walking in children, the relationship between harmony and stability, the principles related to stance/swing ratio that neurophysiological mechanisms follow to optimize gait harmony that has been here just hypothesized, the pathological mechanisms altering this proportion, and possible neurorehabilitation interventions for restoring the stance to swing ratio.

In conclusion, our results place gait phases in a strict relationship with harmonic properties of walking demonstrating that the repetitive gait phases are in turn in repetitive proportions with each other, probably facilitating the control of locomotion. Our study ascribes human gait in those seemingly disparate fields of nature in which harmony acts as a robust unifying factor [18, 29].

## Figures and Tables

**Table 1:** Spatiotemporal gait parameters.

Parameter	Mean ± SD	CV
Walking speed [m/s]	1.14 ± 0.16	13.61%
Stride length [m]	1.29 ± 0.11	8.90%
Stride duration [s]	1.14 ± 0.10	8.68%
Stance phase (foot off) [%]	61.81 ± 2.32	3.75%
Swing phase [%]	38.19 ± 2.31	6.04%
Double support phases [%]	23.62 ± 4.64	19.62%
Opposite foot off [%]	11.80 ± 2.34	19.81%
Opposite foot strike [%]	50.08 ± 0.78	1.55%

Mean ± standard deviation (SD) and coefficient of variation (CV) for the computed values.

## References

[1] Menz HB, Lord SR, Fitzpatrick RC (2003). Acceleration patterns of the head and pelvis when walking on level and irregular surfaces. *Gait and Posture*.

[2] Bruijn SM, Meyns P, Jonkers I, Kaat D, Duysens J (2011). Control of angular momentum during walking in children with cerebral palsy. *Research in Developmental Disabilities*.

[3] Iosa M, Marro T, Paolucci S, Morelli D (2012). Stability and harmony of gait in children with cerebral palsy. *Research in Developmental Disabilities*.

[4] Borghese NA, Bianchi L, Lacquaniti F (1996). Kinematic deferminants of human locomotion. *Journal of Physiology*.

[5] Reisman DS, Block HJ, Bastian AJ (2005). Interlimb coordination during locomotion: what can be adapted and stored?. *Journal of Neurophysiology*.

[6] Cappozzo A (1982). Low frequency self-generated vibration during ambulation in normal men. *Journal of Biomechanics*.

[7] Iosa M, Mazzà C, Frusciante R (2007). Mobility assessment of patients with facioscapulohumeral dystrophy. *Clinical Biomechanics*.

[8] Giakas G, Baltzopoulos V (1997). Time and frequency domain analysis of ground reaction forces during walking: an investigation of variability and symmetry. *Gait and Posture*.

[9] Lamoth CJC, Meijer OG, Wuisman PIJM, van Dieën JH, Levin MF, Beek PJ (2002). Pelvis-thorax coordination in the transverse plane during walking in persons with nonspecific low back pain. *Spine*.

[10] Perry J (1992). *Gait Analysis: Normal and Pathological Function*.

[11] Kirtley C (2006). *Clinical Gait Analysis; Theory and Practice*.

[12] Winter DA, Patla AE, Frank JS, Walt SE (1990). Biomechanical walking pattern changes in the fit and healthy elderly. *Physical Therapy*.

[13] Shemmell J, Johansson J, Portra V, Gottlieb GL, Thomas JS, Corcos DM (2007). Control of interjoint coordination during the swing phase of normal gait at different speeds. *Journal of NeuroEngineering and Rehabilitation*.

[14] Riener R, Rabuffetti M, Frigo C (2002). Stair ascent and descent at different inclinations. *Gait and Posture*.

[15] Wright RB, Yoder DM, Costa JL, Andriacchi TP (1995). Characterization of gait parameters in adult-onset myotonic dystrophy: abnormal hip motion. *Archives of Physical Medicine and Rehabilitation*.

[16] Abernethy B, Hanrahan SJ, Kippers V, Mackinnon LT, Pandy MG (2005). *The Biophysical Foundations of Human Movement*.

[17] Lythgo N, Wilson C, Galea M (2011). Basic gait and symmetry measures for primary school-aged children and young adults. II: walking at slow, free and fast speed. *Gait and Posture*.

[18] Grattan Guinnes I (2003). *Companion Encyclopedia of the History and Philosophy of the Mathematical Sciences*.

[19] Hemenway P (2005). *Divine Proportion: Phi in Art, Nature, and Science*.

[20] Pacioli L (1509). *De Divina Proportione*.

[21] Okabe T (2011). Physical phenomenology of phyllotaxis. *Journal of Theoretical Biology*.

[22] Gosling E (2003). *Bivalve Molluscs. Biology, Ecology and Culture*.

[23] Coldea R, Tennant DA, Wheeler EM (2010). Quantum criticality in an ising chain: experimental evidence for emergent e8 symmetry. *Science*.

[24] Yamagishi MEB, Shimabukuro AI (2008). Nucleotide frequencies in human genome and Fibonacci numbers. *Bulletin of Mathematical Biology*.

[25] Staff L, Hurd P, Reale L, Seoighe C, Rockwood A (2012). The hidden geometries of the Arabidopsis thaliana epidermis. *Plos ONE*.

[26] Woldenberg MJ, O’Neill MP, Quackenbush LJ, Pentney RJ (1993). Models for growth, decline and regrowth of the dendrites of rat Purkinje cells induced from magnitude and link-length analysis. *Journal of Theoretical Biology*.

[27] Ferring V, Pancherz H (2008). Divine proportions in the growing face. *American Journal of Orthodontics and Dentofacial Orthopedics*.

[28] Ricketts RM (1982). Divine proportion in facial esthetics. *Clinics in Plastic Surgery*.

[29] Russell PA (2000). The aesthetics of rectangle proportion: effects of judgment scale and context. *American Journal of Psychology*.

[30] Davis RB, Õunpuu S, Tyburski D, Gage JR (1991). A gait analysis data collection and reduction technique. *Human Movement Science*.

[31] Ounpuu S (1994). The biomechanics of walking and running. *Clinics in Sports Medicine*.

[32] Peppe A, Chiavalon C, Pasqualetti P, Crovato D, Caltagirone C (2007). Does gait analysis quantify motor rehabilitation efficacy in Parkinson’s disease patients?. *Gait and Posture*.

[33] Reynolds NC, Myklebust JB, Prieto TE, Myklebust BM (1999). Analysis of gait abnormalities in Huntington disease. *Archives of Physical Medicine and Rehabilitation*.

[34] Kuan T, Tsou J, Su F (1999). Hemiplegic gait of stroke patients: the effect of using a cane. *Archives of Physical Medicine and Rehabilitation*.

[35] Wang X, Wang Y (2012). Gait analysis of children with spastic hemiplegic cerebral palsy. *Neural Regeneration Research Journal*.

[36] Ivanenko YP, Cappellini G, Dominici N, Poppele RE, Lacquaniti F (2007). Modular control of limb movements during human locomotion. *Journal of Neuroscience*.

[37] Zehr EP (2005). Neural control of rhythmic human movement: the common core hypothesis. *Exercise and Sport Sciences Reviews*.

[38] Ivanenko YP, Poppele RE, Lacquaniti F (2009). Distributed neural networks for controlling human locomotion. Lessons from normal and SCI subjects. *Brain Research Bulletin*.

[39] Graham Brown TG (1911). The intrinsic factors in the act of progression in the mammal. *Proceedings of the Royal Society B*.

[40] McCrea DA, Rybak IA (2008). Organization of mammalian locomotor rhythm and pattern generation. *Brain Research Reviews*.

[41] Orlovsky GN, Deliagina T, Grillner S (1999). *Neuronal Control of Locomotion: From Mollusc to Man*.

[42] Takahashi H, Takada Y, Nagai N, Urano T, Takada A (2000). Serotonergic neurons projecting to hippocampus activate locomotion. *Brain Research*.

[43] Iacono RP, Kuniyoshi SM, Ahlman JR, Zimmerman GJ, Maeda G, Pearlstein RD (1997). Concentrations of indoleamine metabolic intermediates in the ventricular cerebrospinal fluid of advanced Parkinson’s patients with severe postural instability and gait disorders. *Journal of Neural Transmission*.

[44] Cappellini G, Ivanenko YP, Poppele RE, Lacquaniti F (2006). Motor patterns in human walking and running. *Journal of Neurophysiology*.

[45] Dominici N, Ivanenko YP, Cappellini G (2011). Locomotor primitives in newborn babies and their development. *Science*.

[46] Smith SS (1997). Step cycle-related oscillatory properties of inferior olivary neurons recorded in ensembles. *Neuroscience*.

[47] Marlinski V, Nilaweera WU, Zelenin PV, Sirota MG, Beloozerova IN (2012). Signals from the ventrolateral thalamus to the motor cortex during locomotion. *Journal of Neurophysiology*.

[48] Collins SH, Adamczyk PG, Kuo AD (2009). Dynamic arm swinging in human walking. *Proceedings of the Royal Society B*.

[49] Iannarilli F, Vannozzi G, Iosa M, Pesce C, Caprinica L (2013). Effects of task complexity on rhythmic reproduction performance in adults. *Human Movement Science*.

